# L-Plastin Phosphorylation: Possible Regulation by a TNFR1 Signaling Cascade in Osteoclasts

**DOI:** 10.3390/cells10092432

**Published:** 2021-09-15

**Authors:** Meenakshi A. Chellaiah

**Affiliations:** Department of Oncology and Diagnostic Sciences, School of Dentistry, University of Maryland, Baltimore, MD 21201, USA; mchellaiah@umaryland.edu

**Keywords:** L-plastin, phosphorylation, SH2/SH3 domains, Src, TRAF-6, nascent sealing zone

## Abstract

Tumor necrosis factor-alpha (TNF-α) signaling regulates phosphorylation of L-plastin, which is involved in forming the nascent sealing zone, a precursor zone for the matured sealing ring. This study aimed to illustrate the molecular mechanisms of L-plastin phosphorylation and the subsequent formation of the nascent sealing zone in osteoclasts treated with TNF-α. Here, we report that anti-TNF-receptor 1, inhibitors of signaling proteins (Src, PI3-K, Rho, and Rho-kinase), and siRNA of TRAF-6 attenuated the phosphorylation of LPL and filamentous actin content significantly in the presence of TNF-α. An inhibitor of integrin αvβ3, PKC, or PKA did not inhibit TNF-α-induced L-plastin phosphorylation. Inhibitors of Src and PI3-K and not Rho or Rho-kinase reduced tyrosine phosphorylation of TRAF-6, suggesting that Src and PI3-K regulate TRAF-6 phosphorylation, and Rho and Rho-kinase are downstream of TRAF-6 regulation. Osteoclasts expressing constitutively active or kinase-defective Src proteins were used to determine the role of Src on L-plastin phosphorylation; similarly, the effect of Rho was confirmed by transducing TAT-fused constitutively active (V14) or dominant-negative (N19) Rho proteins into osteoclasts. Pull-down analysis with glutathione S-transferase-fused SH2 and SH3 domains of Src and PI3-K demonstrated coprecipitation of L-plastin and TRAF-6 with the SH3 and SH2 domains of the PI3-K and Src proteins. However, the actual order of the interaction of proteins requires further elucidation; a comprehensive screening should corroborate the initial findings of protein interactions via the SH2/SH3 domains. Ultimately, inhibition of the interaction of proteins with SH2/SH3 could reduce L-plastin phosphorylation and affect NSZ formation and bone resorption in conditions that display osteoclast activation and bone loss.

## 1. Introduction

Osteoclasts are bone-resorbing cells, and bone resorption is their sole function. Osteoclasts adhere to the bone matrix to be resorbed. Adhesion of osteoclasts to the bone matrix induces the formation of a sealing zone or sealing ring, which has been deemed an indicator of osteoclast activation for bone resorption. Several studies have shown the involvement of different pathways in the organization of the sealing ring or zone in osteoclasts. However, the actual target molecule(s) that contribute to actin organization requires further elucidation. Many actin-binding proteins (ABPs) have been shown to stabilize and rearrange actin cytoskeletal organization in response to stimuli or during cell migration and adhesion [1,2,3,4,5,6]. The role of L-plastin (LPL) in the actin-bundling process involved in sealing-ring formation also requires further elucidation.

LPL, also known as fimbrin [1] or cytoskeletal-associated protein (CAP), is a leukocyte-specific actin-bundling protein. Although LPL is present in podosomes of osteoclasts [1], its role in osteoclast function is minimal. There are three isoforms of plastins, which include L-plastin, I-plastin, and T-plastin. LPL is present predominantly in hematopoietic cells. T-plastin is present in solid tissue cells, whereas I-plastin is in the small intestine, colon, and kidney [7]. LPL is considered to be a marker for cancer. Plastins cross-link actin filaments to tight bundles, in addition to their unique functions in the cell systems indicated above [7,8,9,10].

Plastins comprise the following: Ca^2+^ binding sites flanked by EF-hand motifs at the amino-terminal (N-terminal) end; two actin-binding domains (ABD1 and 2), each encompassing two tandem calponin homology (CH) domains at the carboxyl-terminal (C-terminal) end; and the binding of two actin filaments to the spatially close ABDs of plastins, which influences bundling of the actin filaments into tight bundles [11,12]. L-plastin localizes to actin-rich membrane structures involved in locomotion, adhesion, and membrane extensions (e.g., filopodia, lamellipodia) [1,13,14]. So far, L-plastin is the only one of the three plastin isoforms known to have two putative (serine-5 and -7) phosphorylation sites. Phosphorylation of these sites was shown to activate cytoskeleton rearrangements via actin bundling in processes essential for chemotaxis and cell adhesion [9,14,15].

Earlier studies, including our own, elucidated the critical role of integrin αvβ3 signaling in osteoclast sealing-zone or -ring formation and function [5,16,17,18,19,20,21,22,23,24,25]. TNF-α, the proinflammatory cytokine, stimulated osteoclasts’ differentiation and resorptive activity [26,27,28]. However, little is known about the regulation by which TNF-α signaling mediates sealing-ring formation in resorbing osteoclasts. We demonstrated that TNF-α stimulates the assembly of actin aggregates (“denoted as nascent sealing zones (NSZs)”) at the early stage of sealing-ring formation independent of integrin αvβ3 signaling. These NSZs then matured into fully functional mature sealing rings by integrin αvβ3 signaling. Furthermore, osteoclasts incubated with native mice bone particles (60–80 µm size) and TNF-α or RANKL changed the expression and phosphorylation levels of LPL and cortactin in a time-dependent manner. Interestingly, changes in the actin organization on NSZ and sealing-ring formation correlated with the phosphorylation state of LPL and cortactin proteins in osteoclasts plated on dentine slices and treated with TNF-α [25].

Most recently, using the TAT-mediated transduction method, we confirmed the role of LPL in NSZ formation. Transduction of TAT-fused full-length LPL peptide significantly increases the number of NSZs and sealing rings. However, transduction of amino-terminal LPL peptides consisting of the serine-5 and -7 amino acids (AAs) reduces the formation of NSZs and sealing rings [29]. Furthermore, transduction of TAT-fused low-molecular-weight amino-terminal LPL peptides (10 amino acids) containing Ser-5 and Ser-7 AAs attenuated cellular LPL phosphorylation, NSZ, or sealing-ring formation, and osteoclast bone resorption. However, these changes did not occur with LPL peptides substituted for serine-to-alanine residues. We also demonstrated that unsubstituted peptides of LPL had no effects on bone formation by osteoblasts [30].

Our next inquiry regarded the possible signaling mechanism that mediates the phosphorylation of LPL. A low concentration of TNF-α was shown to activate osteoclasts and promote actin ring formation in osteoclasts formed in vitro and also extracted from newborn rats [28]. The actual downstream target(s) within the TNF-α pathway have yet to be elucidated. The tumor necrosis factor receptor (TNFR) associated factors (TRAFs) have been shown to mediate many cellular activities of TNFR and Toll-interleukin (IL) 1/18 families [31]. Several members of the TRAF family, such as TRAF-2, -5, and -6, have been implicated in the signaling pathway mediated by various TNFR family members [31,32,33,34]. TRAF-6/c-Src complex regulates osteoclast cytoskeletal reorganization in response to IL1. TRAF-6 or c-Src deficiency in mice results in a similar osteopetrotic phenotype due to impaired osteoclast function without a change in number. These mice also exhibited defects in tooth eruption [35,36]. TRAF-6 and Src colocalized with F-actin in osteoclasts [37]. Thus, TRAF-6 was identified as a prerequisite molecule for osteoclast activation.

Several studies have shown the involvement of PKA and PKC in the phosphorylation of LPL [13,38,39]. LPL phosphorylation by PKA was identified as a necessary step for integrin activation in leukocytes [13,40]. Different stimuli can potentially trigger other kinases that may eventually regulate LPL phosphorylation [39,40,41,42,43].

This study aimed to obtain insight into the molecular mechanisms underlying the phosphorylation of LPL. We used siRNA of TRAF-6 and various inhibitors (Src (PP2), PI3-K (Wortmannin), protein kinase C (PKC) (Go6983 or Staurosporine), protein kinase A (PKA) (H89), and Rho kinase (Y27632), and αv (RGDS peptide)) to determine possible signaling molecules involved in LPL phosphorylation in osteoclasts treated with TNF-α. We found that the TNF-α/TNFR1 signaling pathway involves an Src–PI3K–TRAF-6–Rho/Rho-kinase axis regulating LPL phosphorylation and NSZ formation.

## 2. Materials and Methods

### 2.1. Mice

C57BL/6 mice (six- to eight-week-old mice) were used for osteoclast preparation. These mice were either bought from Harlan Laboratories or generated in the animal facility of the University of Maryland Dental School. The author followed the guidelines and approval of the Institutional Animal Care and Use Committee in the breeding and maintenance of mice. Marrow cells of long bone were used for the experiments.

### 2.2. Reagents

Antibodies to GAPDH and TNFR1 (TNF-α receptor 1) were purchased from R&D Systems (Minneapolis, MN, USA). Secondary antibodies (Cy2- and Cy3-conjugated) were purchased from Jackson Immunoresearch (West Grove, PA, USA). GAPDH and secondary antibodies for immunoblotting were obtained from GE Healthcare (Chicago, IL, USA). The antibody to phosphoserine (p-Serine) was bought from Abcam Company (Cambridge, MA, USA). The phospho-SRC pTyr418 antibody was purchased from Thermo Fisher Scientific Company (Waltham, MA, USA). Mounting solutions for mounting coverslips were bought from Thomas Scientific (Swedesboro, NJ, USA) or Vector Labs (Burlingame, CA, USA). The exoenzyme C3 transferase enzyme and Rho-GTP binding protein-coupled to GST beads were bought from Cytoskeleton, Inc. (Denver, CO, USA). TRAF-6 siRNA and antibodies to L-plastin, Src, TRAF-6, and GAPDH were purchased from Santa Cruz Biotechnology, Inc. (Santa Cruz, CA, USA). Polyacrylamide gel electrophoresis and protein estimation reagents were bought from Bio-Rad (Hercules, CA, USA). Rhodamine-phalloidin, Protein A-Sepharose, GST-Sepharose 4B beads, inhibitors to PKC (Gö 6983 and Staurosporine), PKA (H-89), and integrin αv (cyclo RGD peptide aka cRGDfV), and all other chemicals were purchased from Sigma (St. Louis, MO, USA).

### 2.3. Preparation of Osteoclasts from Long Bones of Mice

Osteoclasts were generated in vitro using mouse long bone marrow cells as described previously [4,24]. After isolation, cells were resuspended in α-10 medium and cultured with the appropriate concentrations of macrophage colony-stimulating factor (m-CSF-1; 10 ng/mL) and the receptor activator of NF-κB ligand (RANKL; 55–75 ng/mL). After three days in culture, media were replaced with fresh cytokines. TRAP-positive multinucleated osteoclasts were seen from day four onward. These osteoclasts were used for all experiments.

### 2.4. Preparation of Bone Particles and Osteoclast Lysate after Various Treatments

#### 2.4.1. Preparation of Bone Particles

After removing bone marrow cells, the long mouse bones were washed extensively with PBS and placed in ethanol until use, which kept them free of cells inside and muscle tissue outside. Next, long bones were air-dried in the cell culture biological hood and homogenized with a mini blender. Finally, bone particles were sieved, and bone particles between 60 and 80 µm in size were used for experiments.

#### 2.4.2. Treatment with Various Inhibitors and siRNA of TRAF-6

Before the addition of TNF-α, osteoclasts generated with RANKL and m-CSF-1 were washed extensively with a serum-free alpha-minimum essential medium. Osteoclast cultures were subjected to one of the following inhibitors for 60 min: αv inhibitor (5–10 µg/mL media; Sigma; C6581), Rho kinase inhibitor (Y27632, 10 µM), PKA inhibitor (H89, 50 µM), or PKC inhibitor (a pan PKC inhibitor Go6983 (20 nM) or Staurosporine (20 nM)). In addition, some cultures were treated with siRNA of TRAF-6 as described below and previously [23], or neutralizing antibody to TNFR1 (3–5 µg/mL) as described previously [25]. Furthermore, osteoclasts were preincubated with the anti-mouse TNFR1 antibody for 60–90 min before the addition of TNF-α to enhance the neutralization. Subsequently, after treatment with an inhibitor of interest or TNFR1 antibody, osteoclasts were treated with bone particles (100 µg/mL) and mouse TNF-α (20 ng/mL). The incubation was continued for an additional 3–4 h.

#### 2.4.3. Transfection of TRAF-6 siRNA and Control or Scrambled RNAi

Transfection of TRAF-6 siRNA and control or scrambled RNAi (Santa Cruz Biotechnology, Inc., Santa Cruz, CA, USA) was conducted using the streptolysin O permeabilization method as described previously [44]. After incubation for 36–48 h at 37 °C, lysates were subjected to Western blot analysis with an antibody to TRAF-6 to test the transfection efficiency. For experiments, after incubation for 36–48 h at 37 °C, osteoclasts were treated with bone particles (100 µg/mL) and mouse TNF-α (20 ng/mL) for 3–4 h [25].

#### 2.4.4. Infection of Osteoclasts with Adenovirus Containing Src Constructs

Kinase-defective (KD-Src; K295M) and constitutively active Src (CA-Src; Y527F) were generated essentially based on the pAdEasy-1 system. The virus was propagated as described previously [45]. Adenoviruses containing Src constructs were added to osteoclasts at a 10–30 multiplicity of infection in the serum-free medium described previously [44]. Two hours after infection, the medium was replaced with serum (10%) containing α-MEM medium. Expression of Src was evaluated by immunoblotting the lysate with an Src antibody 48–72 h post-infection. PBS-treated osteoclasts were used as controls. After 40–45 h, cultures were washed and combined with bone particles (100 µg/mL media) for 3–4 h in the presence of TNF-α (20 ng/mL).

#### 2.4.5. Transduction of Osteoclasts with TAT-Fused Rho Constructs

Constitutively active (Rho^Val14^) and dominant-negative (Rho^Asn19^) (indicated as V14 and N19) were cloned in-frame into a pTAT–HA vector [46] to produce fusion proteins containing TAT and HA sequences. TAT–HA-fused Rho proteins were purified using a Ni-NTA column as described previously [47]. After cells were kept in serum-free α-MEM for 2 h, osteoclasts were transduced with Rho proteins. Dose- and time-dependent uptake of TAT-fused Rho proteins in osteoclasts demonstrated that maximal uptake and response were seen at a 100–150 nM concentration. In addition, uptake was seen within 15 min of incubation with TAT proteins [47]. Based on these studies [47,48], we used V14 and N19 Rho proteins at a final concentration of 100 nM in serum-free media.

#### 2.4.6. Preparation of Lysates after Various Treatments

Osteoclasts were incubated with bone particles in the presence of TNF-α and the indicated treatments above for 3–4 h before lysate preparations or any other analyses. Then lysate was made with a RIPA lysis buffer as described [49]. Protein estimation was done using a Bio-Rad protein assay reagent (Hercules, CA, USA).

#### 2.4.7. Immunoprecipitation and Immunoblotting Analyses

An equal amount of proteins was used for immunoprecipitation. Immunoprecipitations and immunoblotting were performed as described [44,49].

### 2.5. Purification of GST-Fusion Proteins and GST-Pull-Down Assay

#### 2.5.1. GST-Fusion Proteins

The pGEX vectors containing cDNA sequences encoding the SH2 and SH3 domains of p85, full-length p85, and the SH2 domain of c-Src and Lck were expressed in Escherichia coli as GST-fusion proteins and purified as described previously [50].

#### 2.5.2. Glutathione S-Transferase (GST)—Fusion Pull-Down Analyses

For the pull-down assay, 5 µg of GST-fusion proteins noncovalently coupled to Sepharose beads were incubated for 2 h at 4 °C with 200 µg of lysates made from osteoclasts treated with TNF-α and bone particles for 3–4 h. After binding, the Sepharose beads were washed four to five times with lysis buffer and washed three times with cold PBS. Bound proteins were boiled with SDS-PAGE sample buffer and subjected to 10% SDS-PAGE. Immunoblotting with LPL and TRAF-6 antibodies was conducted after transferring the proteins onto a polyvinylidene difluoride (PVDF) membrane [49]. GST-alone coupled beads were used to detect nonspecific binding to the GST protein.

### 2.6. Immunostaining and Confocal Analysis of Cells Cultured on Dentine Matrix

Osteoclasts (10^5^ cells) were cultured on dentine slices and incubated with TNF-α for 3–4 h and 8 h. Immunostaining was done with TRAF-6 and Src antibodies in osteoclasts plated for 3–4 h and 8 h; and TRAF-6 and TNF-R1 antibodies in osteoclasts cultured for 3–4 h. In addition, some cultures were stained with rhodamine-phalloidin as described previously [51] to determine the distribution of actin in resorbing osteoclasts after various treatments. Finally, immunostaining, imaging in a confocal microscope (Bio-RAD, Hercules, CA, USA), and processing of the images were conducted as described previously [4,47].

### 2.7. Quantification of Filamentous Actin (F-Actin) Content

We measured the F-actin content in osteoclasts incubated with bone particles (100 µg/mL media) for 3–4 h in the presence of TNF-α (20 ng/mL) and treated as indicated in the figure and above. For each treatment, three to four wells of 24-well culture dishes were used. The cells were fixed and stained with rhodamine-phalloidin. In control experiments, a 10-fold excess of unlabeled phalloidin was used to determine the nonspecific binding. The nonspecific binding was subtracted from the total binding to obtain the specific binding. The cells were washed quickly several times with PBS and extracted with absolute methanol. As previously described, the fluorescence was measured using a spectrofluorometer (Bio-RAD, Hercules, CA, USA) [4,49].

### 2.8. Dentine Matrix Resorption Assay

Mature osteoclasts were plated on dentine matrix and allowed to adhere for 2–3 h. Based on previous studies [25], 3–5 µg/mL TNFR1 antibody was used, and incubation was continued for 14–18 h in the presence of TNF-α (20 ng/mL). To improve the neutralization effect, TNF-α was added 60–90 min after adding the antibody. To determine the TRAF-6 knockdown effect on dentine resorption, mature osteoclasts transfected with TRAF-6 siRNA and scrambled RNAi were plated on dentine slices and allowed to adhere for 2–3 h. Then, TNF-α was added to all cultures, and incubation was continued for 48–72 h. Each treatment was done in triplicate or quadruplicate. Subsequently, dentine slices were processed and stained with Meyer’s acid hematoxylin (Sigma, St. Louis, MO, USA). Pits were imaged in a Zeiss phase-contrast microscope (40× objective) fitted with a SPOT camera (Diagnostic Instruments, Alexandria, VA, USA) and processed as previously described [52].

### 2.9. Statistical Analysis

All values were mean ± SEM of three or more experiments performed at different times with different osteoclast preparations. A Student’s *t*-test or two-way ANOVA (Graph Pad Software from Graph Pad Inc., San Diego, CA, USA) was used to determine the statistical significance. A *p* < 0.05 was considered significant.

## 3. Results

LPL phosphorylation and the formation of actin aggregates known as NSZs were observed within 3–4 h of treatment of TNF-α and bone particles [25]. Therefore, we determined the causative signaling molecules/cascade involved in LPL phosphorylation during this period and their possible interactions. Various treatments were applied to determine the potential signaling pathway. Osteoclasts that were not treated but were incubated with TNF-α and bone particles for 3–4 h were used as untreated controls (indicated as untreated) in studies shown below unless otherwise mentioned).

### 3.1. Immunoblotting Analysis of Phosphorylation of TRAF-6 in Osteoclasts Treated with TNF-α

**Figure 1:**
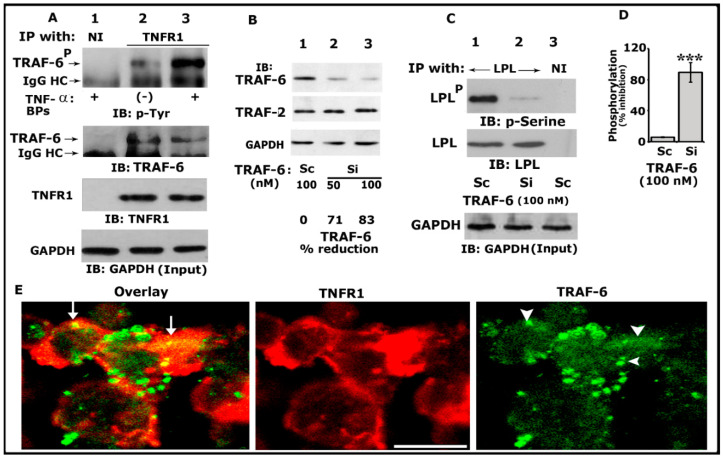
Analysis of the phosphorylation of TRAF-6 by immunoblotting and interaction of TRAF-6 with TNFR1. An equal amount of lysate proteins was used for the immunoblotting (IB) analyses shown in panels (**A**–**C**). IB analyses were performed sequentially with the indicated antibody after stripping the blot shown in each panel (**A**–**C**). An equal amount of total protein used for immunoprecipitation was assessed by direct IB of the lysates (Input) with a GAPDH antibody (**A**,**C**, bottom panels). (**A**) IB analysis of the interaction of TRAF-6 with TNFR1. Osteoclasts untreated (indicated by a minus sign) or treated (indicated by plus signs) with TNF-α and bone particles for 3–4 h were immunoprecipitated with an antibody to TNFR1 (lanes 2 and 3) or nonimmune serum (NI; lane 1). IB analyses were performed sequentially with indicated antibodies. Phosphorylated TRAF-6 is indicated as TRAF-6^P^ (**B**). The effects of siRNA of TRAF-6 on the cellular levels of TRAF-6 and TRAF2. An equal amount of total lysate made from osteoclasts treated with a siRNA (Si) or scrambled or RNAi (Sc) was used for the indicated IB analyses in (**B**). GAPDH was used as a loading control (bottom). The percent reduction of TRAF-6 protein for the representative blot is provided below the panel. The experiment was repeated thrice and obtained a comparable reduction. (**C**) The effects of siRNA of TRAF-6 on the phosphorylation of LPL. An equal amount of lysate proteins was immunoprecipitated with an antibody to LPL (lanes 1 and 2) or nonimmune serum (lane 3). IB analyses were performed with the indicated antibodies in the figure. Phosphorylated LPL is indicated as LPL^P^ (**D**). Percent inhibition of LPL phosphorylation is provided in the graph. *** *p* < 0.001 versus control RNAi (**C**) treated osteoclasts. Values were expressed as mean ± SEM (*n* = 3). Uncropped images of autoradiograms (**A**–**C**) are provided in the Supplementary Materials. (**E**) Confocal analysis of the localization of TRAF-6 and TNFR1 in osteoclasts plated on dentine slices in the presence of TNF-α for 2–3 h. The yellow color (indicated by arrows) in the overlay panel represents the colocalization of TRAF-6 with TNFR1. Arrowheads in the green panel point to TRAF-6 localization, where colocalization is shown in the overlay panel. Scale bar: 100 µm.

TRAFs have been implicated in the signaling processes mediated by TNF receptor family members, including TNFR1, TNFR2, CD30, and CD40 [53]. In addition, TRAF-6 has been implicated in the cytoskeletal organization and resorptive function of osteoclasts in vitro [54]. We have previously shown that TNF-α/TNFR1 signaling regulates the phosphorylation of LPL and actin-bundling processes involved in bone resorption [25,29]. Thus, we first sought to determine whether TRAF-6 could be one of the downstream regulators of TNF-α/TNFR1 signaling. Osteoclast lysates made from TNF-α untreated (indicated with a minus sign) or treated (+) cells were immunoprecipitated with an antibody to TNFR1 (Figure 1A, lanes 2 and 3) or nonimmune serum (NI; lane 1). Our initial analyses indicated that TRAF-6 (~MW 58–60 kDa) was not only coprecipitated with TNFR1 but also phosphorylated more in response to TNF-α (lane 3). Immunoblotting analysis with TRAF-6 and TNFR1 antibodies demonstrated the immunoprecipitated protein levels of TRAF-6 and TNFR1 (Figure 1A, middle panels).

Next, to determine the requirement of TRAF-6 in LPL phosphorylation, we used the RNA interference (RNAi) strategy to reduce TRAF-6 protein levels. We used siRNA-mediated silencing of TRAF-6 at doses of 50 and 100 nM for 36–48 h at 37 °C. A dose-dependent decrease in the level of TRAF-6 was observed at 50 and 100 nM siRNA. The reduction was significant (>80%) at a dose of 100 nM (Figure 1B, top panel, lane 3). Osteoclasts transfected with a scrambled RNAi (indicated as Sc were used to control siRNA-mediated effects (Figure 1B, lane 1). The effect of TRAF-6 siRNA was found to be more specific and effectively reduced only TRAF-6 and not TRAF 2 (Figure 1B, middle panel). Therefore, 100 nM of TRAF-6 siRNA was used in the following experiments. The GAPDH immunoblot demonstrates the total lysate proteins used for immunoblotting analysis (bottom panel).

Subsequently, equal amounts of protein lysates were used for immunoprecipitation with an LPL antibody and immunoblotted with a p-serine antibody (Figure 1C; Top panel). A significant decrease (>85%; Figure 1C, lane 2; Figure 1D) in the phosphorylation of LPL was observed in osteoclasts transfected with TRAF-6 siRNA, which suggested that TRAF-6 is one of the regulators of phosphorylation of LPL. Scrambled RNAi-treated cells were used as controls (Figure 1C, lane 1; Figure 1D). Stripping and reblotting of the same blot with an LPL antibody demonstrated the amounts of LPL in each immunoprecipitate (Figure 1C, middle panel). A GAPDH immunoblot was used to show the amount of protein used for the immunoprecipitations (Figure 1A,C, bottom panel).

Confocal microscopy analyses of osteoclasts plated on dentine slices for 3–4 h (Figure 1E) demonstrated dense TNFR1 (red) distribution near the plasma membrane. In these dense areas, colocalization (yellow color, indicated by arrows) of TNFR1 and TRAF-6 (green) was observed. Although the punctate and diffuse distribution of TRAF-6 was observed in osteoclasts, colocalization was observed only in areas where the dense distribution of TNFR1 was observed (overlay panel; indicated by arrows). These areas were prospective NSZs. Thus, coprecipitation (Figure 1A) and colocalization of TRAF-6 with TNFR1 do not mean that these molecules are physically associated with each other. Instead, it suggests the possibility that they are interacting with each other.

### 3.2. Analysis of TNF-α-Mediated LPL Phosphorylation in Response to Various Treatments

**Figure 2:**
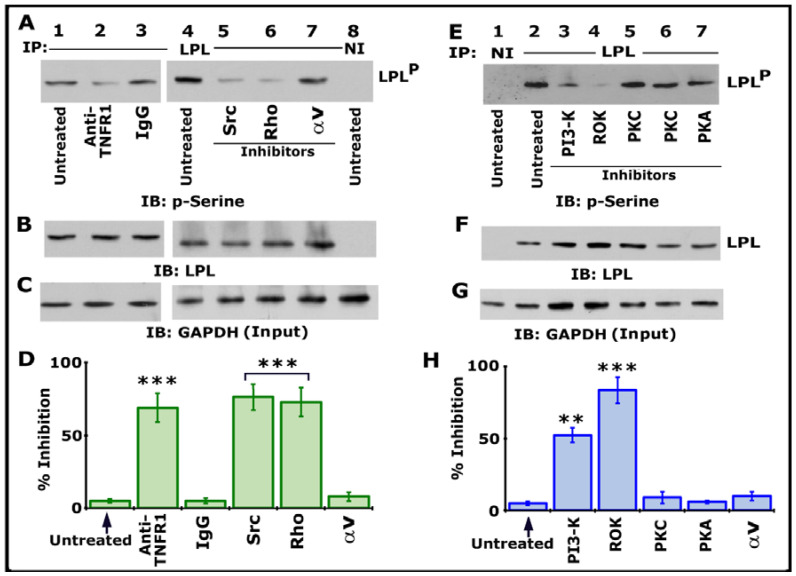
Immunoblotting (IB) analyses in lysates made from osteoclasts treated with various treatments. Osteoclasts untreated (lanes 1 and 4) or treated with IgG (lane 3) were used as controls. All cultures were incubated with TNF-α and bone particles for 3 h. (**A**–**C**) Lysates made from osteoclasts treated with anti-TNFR1 (lane 2); species-specific IgG (lane 3); or inhibitors of Src (PP2), Rho (C3), or integrin αv (cyclo RGD peptide) (lanes 5–7) were immunoprecipitated with an antibody to LPL (**A**, 1–7) or nonimmune serum (NI; lane 8) for IB analysis with a p-serine antibody (**A**). LPL^P^ represents phosphorylated LPL. Then, the blot was stripped and immunoblotted with an antibody to LPL to determine the immunoprecipitated levels of LPL (**B**). Finally, direct immunoblotting of the lysates with a GAPDH antibody in (**C**) demonstrated that an equal amount of total protein (Input) was used for immunoprecipitation with LPL (**C**). (**E**–**G**) Lysates made from osteoclasts treated with inhibitors of PI-3K (Wortmannin), Rho kinase (Y27632), PKC (Staurosporine or Go6983), or PKA (H89) (lanes 3–7) were immunoprecipitated with an antibody to LPL (**E**, 2–7) or nonimmune serum (NI; lane 1) for IB analysis with a p-serine antibody (**E**). The blot was then stripped and immunoblotted with an antibody to LPL to determine the immunoprecipitated levels of LPL (**F**). Immunoblotting of the lysates with a GAPDH antibody in panel G indicates the total protein (Input) used for immunoprecipitation with LPL (**C**). (**D**,**H**) Percent inhibition of phosphorylation of LPL (~68–70 kDa) is provided as a graph. The resulting LPL^P^-to-LPL ratio was used for the quantitative analyses. *** *p* < 0.001; ** *p* < 0.01 versus untreated osteoclasts. Values shown are mean ± SEM of three different immunoblots. The results shown are one of the three experiments performed with similar results. Uncropped images are provided in the Supplementary Materials.

We then assessed the possible TNF-α- and TNFR1-mediated signaling axis using various inhibitors targeting Src, PI3-K, PKC, PKA, Rho, and Rho kinase (Figure 2). Osteoclast cultures not treated with any inhibitor (indicated as untreated) were used as controls. All cultures were treated with TNF-α and bone particles for 3–4 h after respective inhibitor treatment as described in Section 2. Lysates were immunoprecipitated with an LPL antibody or species-specific nonimmune IgG (NI) (Figure 1A, lane 8; Figure 1E, lane 1). Immunoblotting was done with a p-serine antibody. Inhibiting LPL phosphorylation with a neutralizing antibody to TNFR1 (Figure 2A, lane 2) validated our previous observation [25]. Antibody-untreated and species-specific IgG-treated osteoclasts were used as controls (Figure 2A, lanes 1 and 3). A significant reduction in the phosphorylation of LPL was observed in cells treated with inhibitors of Src (PP2), Rho (C3 exoenzyme), PI3-K (Wortmannin, abbreviated as WM), and Rho kinase (abbreviated as ROK, Y27632) (Figure 2A, lanes 5 and 6; Figure 2E, lanes 3 and 4). LPL phosphorylation was unaffected in cells treated with inhibitors of αv (cyclo RGD peptide; Figure 2A, lane 7), PKC (Go6983 or Staurosporine), and PKA (H89) (Figure 2E, lanes 5–7). Two different PKC inhibitors were used. Stripping and reblotting of the p-serine-probed blot demonstrated the immunoprecipitated levels of LPL. Immunoblotting of total cellular lysates with a GAPDH antibody presented the amount of lysate (Input) used for the immunoprecipitation (C and G). Statistical analysis of phosphorylation of LPL from three different experiments is provided as a graph (Figure 2D,H). Percent inhibition was significant with inhibitors of Src, PI3-K, Rho, and ROK. As consistently shown previously [25], an inhibitor of αv did not affect LPL phosphorylation, suggesting that this occurs independently of integrin signaling. It was TNF-α and TNFR1-mediated phosphorylation of LPL, an event that involves signaling proteins such as Src–PI3-K–TRAF-6–Rho–Rho-kinase. The exact order of the signaling axis in LPL phosphorylation requires further elucidation.

### 3.3. Analysis of Phosphorylation of LPL in Osteoclasts Expressing Src or Transduced with Rho Proteins

To further elucidate the role of Src in LPL phosphorylation, osteoclasts were infected with adenovirus containing constitutively active (CA) and kinase-defective (KD) Src constructs. Immunoblotting analysis with an Src antibody exhibited more CA and KD-Src proteins (Figure S1A, lanes 2 and 3) than cellular Src level (lane 1). Phosphorylation of LPL (Figure S1C) corresponded with the expression levels of cellular (lane 1) and CA-Src proteins (Figure S1C, lanes 1 and 3). Although the expression level of KD-Src was equal to CA-Src (Figure S1A, lanes 2 and 3) and more than the control (lane 1), LPL phosphorylation was below the control levels. Stripping and reprobing the same blot elucidated that those comparable levels of L-plastin were immunoprecipitated (Figure S1H).

To confirm the role of Rho as an upstream regulator of LPL phosphorylation, osteoclasts were transduced with constitutively active (V14) and dominant-negative (N19) Rho proteins as described previously [5,47,48]. Maximum uptake of TAT-fused Rho proteins took place after 40–60 min, and the uptake decreased after two hours. However, the stability of the transduced proteins lasted for up to 6 h and declined from 12 h [5]. Therefore, transduction of Rho proteins (V14 and N19) was conducted for 45 min, and then cells were treated with TNF-α and bone particles for 3–4 h. Transduced proteins had an HA tag; therefore, an HA antibody was used for immunoblotting analysis. Immunoblotting analysis with a Rho and HA antibody displayed the transduced levels of Rho proteins (Figure S2A,C, lanes 2 and 3). Untransduced control cells but treated with TNF-α and bone particles for 3–4 h are shown in lane 1.

Then, we measured the GTP-Rho levels in untransduced (-) and Rho-protein-transduced (V14 and N19) cells (Figure S2D, lanes 1–3). GST-fused Rho-binding domain (RBD) was used to detect the GTP-bound Rho proteins. After binding, beads were washed. Then, the bound proteins were subjected to immunoblotting analysis with a Rho antibody that demonstrated retainment of transduced TAT-V14 Rho (Figure S2D, lane 3) and cellular Rho protein (lanes 1–3) to GST-RBD-coupled glutathione beads. Therefore, these were considered GTP-bound Rho and indicated as TAT-Rho-GTP and Rho-GTP (Figure S2D). On the other hand, no detectable TAT-N19-Rho was held to GST-RBD, which possibly meant the GDP-bound form. Figure S2E displays the amount of Rho (transduced and cellular) present in the cellular lysate. In addition, the GAPDH immunoblot demonstrated the amount of lysate protein used (Input) for the GST-GBD binding studies (panel F).

Immunoblotting of LPL immunoprecipitates with a p-serine antibody exhibited more LPL phosphorylation in osteoclasts transduced with V14 Rho (Figure S2G, lane 3) than N19 Rho-transduced cells (panel G, lane 2) and untransduced cells ((-), lane 1). The phosphorylation of LPL was lower in N19 Rho-transduced cells (panel G, lane 2) than in untransduced cells, although the Rho-GTP level was equal in these cells (panel D, lanes 1 and 2). Stripping and reprobing of the blot with an LPL antibody revealed the amount of LPL in each immunoprecipitate. These data suggested that TNF-α/TNFR1 signaling, which involves the activation of Src and Rho proteins, regulates the phosphorylation of LPL.

### 3.4. TNF-α Regulates TRAF-6 Phosphorylation via a Pathway That Involves Src and PI3-K

**Figure 3:**
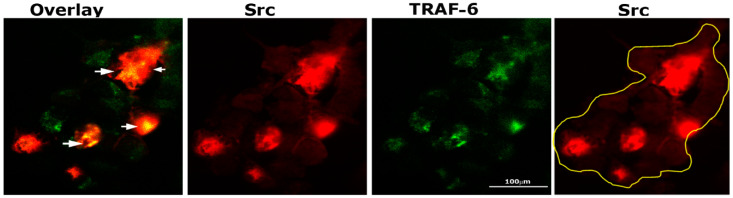
Immunostaining and confocal analyses of Src (red) and TRAF-6 (green) localization in osteoclasts plated on dentine slices and treated with TNF-α for 2–3 h Colocalization is indicated by arrows in overlay panels. The osteoclast is outlined with a yellow line in the Src (red) panel. The experiment was repeated twice, and similar results were obtained. Scale bar: 100 µm.

TRAF-6 and Src knockout mice develop osteopetrosis due to impaired bone resorption by osteoclasts [35,36]. In addition, cytoskeletal organization by IL-1 is mediated by the TRAF-6/Src complex [37]. Therefore, we sought to determine by immunostaining analysis whether Src (red) and TRAF-6 (green) were colocalized in osteoclasts plated on dentine and treated with TNF-α for 3–4 h (Figure 3). Intense staining of Src was observed in dense patches, and in some of those dense areas, colocalization (yellow color) of TRAF-6 and Src was found (overlay panel; indicated by arrows). Immunostaining analysis demonstrated possible interaction between these two proteins.

**Figure 4:**
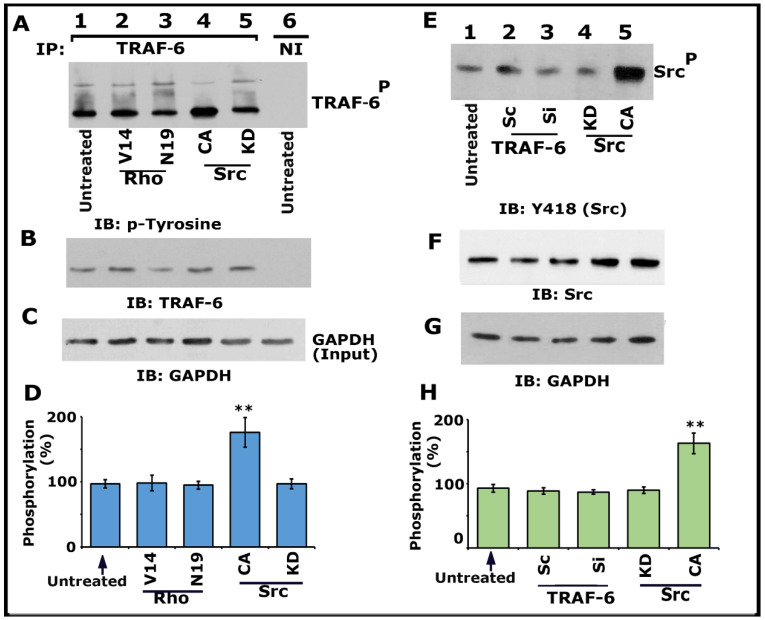
Immunoblotting IIB) analysis of phosphorylation of TRAF-6 and Src kinase on Y418. Osteoclasts untreated and treated with indicated treatments were incubated with TNF-α and bone particles for 3–4 h. (**A**–**D**) Lysates made from osteoclasts untreated (lanes 1 and 6) and either transduced with TAT-Rho proteins (V14 and N19; lanes 2 and 3) or transfected with Src Ad-Src constructs (CA and KD; lanes 4 and 5) were immunoprecipitated with a TRAF-6 antibody or nonimmune serum (NI; lane 6). IB was performed with a p-tyrosine antibody (**A**). The blot was stripped and immunoblotted with an antibody to TRAF-6 to determine the immunoprecipitated levels of TRAF-6 (**B**). Lastly, direct immunoblotting of the lysates with a GAPDH antibody in panel C demonstrates the equal amount of total protein (Input) was used for immunoprecipitation with TRAF-6 (**C**). Percent phosphorylation of TRAF-6 is provided as a graph (**D**). ** *p* < 0.01 versus untreated osteoclasts. Values are the mean ± SEM of three different immunoblots. (**E**–**H**) Lysates made from these osteoclasts treated as indicated in panel E were immunoblotted with an antibody to Tyrosine 418 (Src) (**E**). The blot was stripped in sequence and immunoblotted respectively with an Src and then with a GAPDH antibody as loading controls for the result shown in panel E. The resulting pTRAF-6/TRAF-6 ratio was used for the quantitative analyses. ** *p* < 0.01 versus untreated osteoclasts. Results represent one of the three experiments with similar findings. Uncropped images are provided in the Supplementary Materials.

Having found that Src, PI3-K, TRAF-6, and Rho kinase regulate the phosphorylation of LPL (Figure 1 and Figure 3; Figures S1 and S2), we then asked whether Src and Rho are upstream regulators of TRAF-6. Immunoprecipitates made with a TRAF-6 antibody were immunoblotted with a phosphotyrosine (p-tyrosine) antibody (Figure 4A). A significant increase in TRAF-6 phosphorylation was observed in osteoclasts expressing CA-Src (Figure 4A, lane 4; Figure 4D), but not in osteoclasts expressing KD-Src (lane 5). In addition, basal level phosphorylation was observed in the untreated control (lane 1) and Rho protein (V14 and N19)-transduced (Figure 4A, lanes 2 and 3; Figure 4D) osteoclasts.

Subsequently, we confirmed that TRAF-6 did not regulate Src phosphorylation on Y418 using the lysates made from cells treated with siRNA and ScRNAi to TRAF-6. Neither siRNA nor ScRNAi affected the phosphorylation of Src on Y418. Although an increase in Src Y418 phosphorylation was observed in cells transfected with CA-Src (Figure 4E, lane 5), other treatments (lanes 2–4) displayed basal level phosphorylation of Src as observed in untreated cells (Figure 4E, lane 1). Next, we investigated the effect of Src, PI3-K, Rho, and Rho-kinase inhibitors on TRAF-6 phosphorylation (Figure S3). As projected, inhibitors of Src (Figure S3A, lane 4) and PI3-K (Figure S3A, lane 3; Figure S3B, lane 5) reduced the phosphorylation of TRAF-6. In contrast, Rho and rho kinase inhibitors did not affect the phosphorylation of TRAF-6 (Figure S3A, lane 2; Figure S3B, lanes 3 and 4). Basal level phosphorylation of TRAF-6 was observed in untreated osteoclasts (Figure S3A, lane 1; Figure S3B, lane 2). Lysates from these osteoclasts were also used for nonimmune (NI) immunoprecipitation (Figure S3B, lane 1). Subsequently, the blot was stripped and immunoblotted with an antibody to LPL to determine the immunoprecipitated levels of LPL (F). Direct immunoblotting of the lysates with a GAPDH antibody in panel G indicates the total protein (Input) used for immunoprecipitation with LPL (C). Percent inhibition of TRAF-6 phosphorylation is provided as a graph.

Collectively, our data provided evidence that TRAF-6 does not mediate Src phosphorylation. An increase in the phosphorylation of TRAF-6 in osteoclasts expressing CA-Src implied that Src is upstream of TRAF-6 in the signaling pathway, which regulates LPL phosphorylation. Since V14Rho did not affect the phosphorylation of TRAF-6, we suggest it may be a downstream regulator of Src and TRAF-6. Based on these studies (Figure 4 and Figure S3), we recommend that the TNF-α/TNFR1 signaling axis of Src–PI3-K–TRAF-6–Rho/Rho-kinase proteins regulate the phosphorylation of LPL. 

### 3.5. GST-Fusion Pull-Down Assay

**Figure 5:**
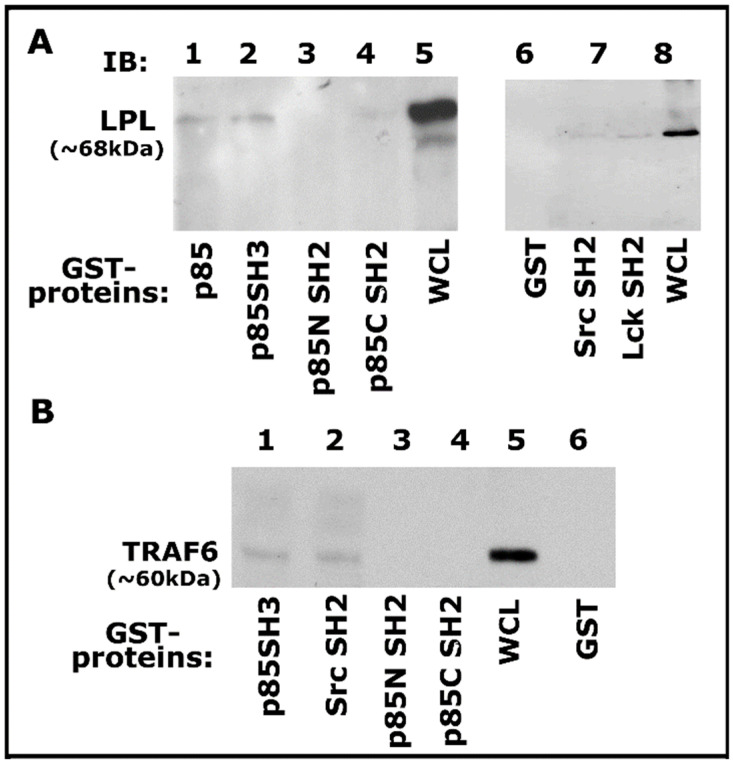
Analysis of binding of proteins (LPL and TRAF-6) with indicated GST-fusion proteins by GST pull-down assay. (**A**,**B**) Analysis of the specificity of binding of LPL (**A**) and TRAF-6 (**B**) with indicated domains of signaling proteins by GST pull-down assay. Lysate (100 µg) made from osteoclasts treated with TNF-α and bone particles for 3–4 h were used for this assay. The binding of LPL (**A**; ~68–70 kDa) or TRAF-6 (**B**; ~60 kDa) with the GST-fused protein of interest was determined by immunoblotting with the corresponding antibody. Whole-cell lysate (WCL) protein (~25 µg) was used to identify LPL (**A**, lanes 5 and 8) and TRAF-6 (lane 5 in **B**) proteins. Pull-down with GST (vector protein)-coupled glutathione Sepharose beads was used to determine nonspecific binding (**A**,**B**, lane 6). The experiment was repeated twice, and similar results were obtained. Uncropped autoradiogram images are provided in this figure.

Then we proceeded to determine the binding specificity of TRAF-6 with Src and PI3-K using GST-fused SH2 and SH3 domains of Src, Lck, and PI3-K proteins coupled to Sepharose beads as shown previously [55]. Lysates made from osteoclasts treated with TNF-α and bone particles for 3–4 h were used for this GST pull-down analysis. Pull-down of LPL was observed with GST-fused full-length PI3-K (p85), p85 SH3 domains (Figure 5A, lanes 3 and 4), and not with p85 amino- (p85N SH2) and carboxyl- (p85C SH2) terminal SH2 domains of p85 (Figure 5A, lanes 3 and 4). However, LPL pull-down was also observed with Src and Lck SH2 domains, but to a lesser extent (Figure 5A, lanes 6 and 7). In addition, an equal binding of TRAF-6 to the p85-SH3 and Src-SH2 domains was observed (Figure 5B, lanes 1 and 2). In the binding studies, the whole-cell lysate (WCL) was used to detect LPL and TRAF-6 as markers for pull-down proteins (Figure 5A,B). Our results suggested that a complex interaction may occur between these proteins via the SH2 and SH3 domains. Pull-down analysis reinforced the contribution of these signaling proteins. However, the actual mechanism of interaction requires further elucidation.

### 3.6. Analysis of Actin Distribution and F-Actin Content in Osteoclasts Subjected to Various Treatments

**Figure 6:**
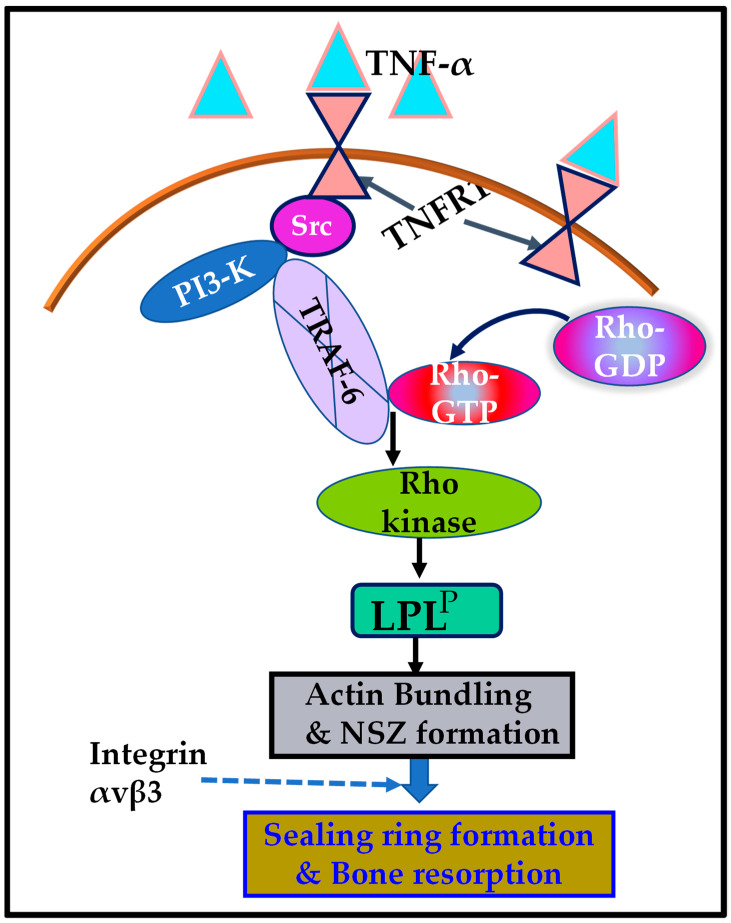
Schematic representation of the regulation of phosphorylation of L-plastin (LPL) by the signaling mechanism mediated by TNF-α/TNFR1. The present study suggested that the TNF-α/TNFR1 signaling pathway, which involves an Src/PI3-K/TRAF-6/Rho kinase axis, regulates the L plastin phosphorylation (LPL^P^) and actin-bundling process needed for nascent sealing zone (NSZ) formation. Subsequently, the localization of integrin αvβ3 in NSZs regulates their maturation of NSZs into fully functional mature sealing rings, which are vital for bone resorption mediated by osteoclasts attached to the bone surface.

Osteoclasts cultured on dentine slices in the presence of TNF-α for 3–4 h demonstrated the formation of NSZs. Here, we examined the effect of inhibitors of Src, Rho-kinase (ROK), PI3-K, and SiRNA of TRAF-6, on actin distribution (Figure S4A–E). Consistently, as shown previously [25,29,30,56,57,58], inhibitor-untreated osteoclasts but treated with TNF-α for 3–4 h displayed several NSZs (Figure S4A; indicated by arrows). NSZs were not seen in osteoclasts treated with inhibitors of Src, ROK, or PI3-K, or SiRNA to TRAF-f. Small patchy and punctate distribution of actin was observed in osteoclasts treated with inhibitors of Src, PI3-K, and ROK (Figure S4B–D). F-actin measurement demonstrated a significant decline in osteoclasts treated with inhibitors of Src, PI3-K, and ROK, as well as siRNA of TRAF-6. F-actin content was reduced in osteoclasts that failed to form NSZs. F-actin level in scrambled RNAi (Sc) or an inhibitor αv and PKC- treated osteoclasts was equal to untreated osteoclasts (Figure S4F). A decrease in the F-actin content, the presence of punctate structures, and failure to form NSZs in osteoclasts treated with inhibitors of Src, PI3-K, and ROK, as well as siRNA of TRAF-6, substantiated their role in the actin-bundling process mediated by LPL (schematic diagram in Figure 6).

### 3.7. Dentine Matrix Resorption Assay

**Figure 7:**
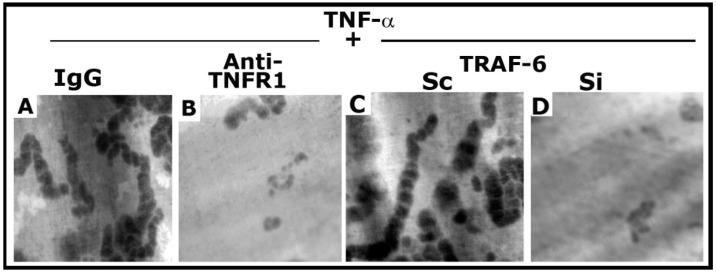
The effect of anti-TNFR1 and TRAF-6 SiRNA on osteoclast bone resorption in vitro. Resorption pits were seen as dark spots. Resorption pits were viewed under a 40× objective in a phase-contrast microscope and photographed. The magnification was ×400. For each treatment, three to four dentine slices were used per experiment. Experiments were repeated two times with two different osteoclast preparations, and similar results were obtained. Treatments were as follows: IgG: Species-specific IgG control for Anti-TNFR1 (**A**); Anti-TNFR1: antibody to TNFR1-treated osteoclasts (**B**); Scrambled RNAi (Sc)-treated osteoclasts (**C**); Si: SiRNA of TRAF-6 treated osteoclasts (**D**).

As shown previously [25], anti-TNFR1 significantly reduced the resorption elicited by TNF-α (Figure 7B). In addition, TRAF-6 siRNA exhibited a similar inhibitory effect on the resorption activity induced by TNF-α (Figure 7D). However, control osteoclasts (IgG and scrambled RNAi transfected) revealed the formation of multilocular resorption pits in response to TNF-α treatment. Thus, inhibition of NSZ formation by the neutralizing antibody to TNFR1 [25] and siRNA of TRAF-6 (Figure S4) corresponded with decreased bone resorption activity of osteoclasts.

## 4. Discussion

L-plastin (LPL) phosphorylation on serine-5 and -7 residues by TNF-alpha signaling regulates the actin-bundling process by LPL and, hence, the assembly of NSZs in mice osteoclasts. We confirmed the role of phosphorylation of Ser-5 and Ser-7 in actin-bundling by using mutated (A5A7) full-length and low-molecular-weight LPL peptides [29,30,56]. Furthermore, studies have demonstrated the role of LPL expression and phosphorylation in prostate cancer cells on invasion in vitro and tumor growth and metastasis in vivo in a mouse model [59,60]. Although studies have shown that several kinases participate in LPL phosphorylation, the actual mechanism is unknown (reviewed in Schaffner-Reckinger et al. [61]). This study has revealed that the TNF-α/TNFR1 signaling cascade that involves Src–PI3–Kinase–TRAF-6–Rho/Rho-kinase regulates the phosphorylation of LPL and promotes NSZs formation.

TRAFs (TRAF-2, -5, and -6) have been implicated in regulating signals from various TNF-receptor family members, leading to NF-kB activation [32,34,62,63,64,65,66,67]. In addition, studies have shown that TRAF-6 links several families of cytokine receptors to the activation of NF-kB [68,69,70]. However, it has been debated whether TRAF-6 is essential for osteoclast formation. Two groups have generated knockout mice for TRAF-6; one showed that TRAF-6 was necessary for osteoclastogenesis [71], and the other for osteoclast function [35]. Furthermore, studies with mutant TRAF-6 in the RANK-binding domain demonstrated that TRAF-6 is involved in, but not essential to, osteoclastogenesis [72]. Therefore, it was suggested that the signals leading to osteoclast differentiation could be counterbalanced by other TRAFs (TRAF-2 and/or TRAF-5). However, TRAF-6 is crucial in mature osteoclasts for its bone resorption function [35]. Using the siRNA strategy and bone resorption assay in vitro, studies were shown here that TRAF-6-mediated signaling has a role in cytoskeletal modulation required for osteoclast function.

TRAF-6 was shown to be a required molecule in the formation of cytoskeletal structures and the resorptive activity of osteoclasts [54]. This paper has demonstrated that mouse osteoclasts incubated with native bone particles and TNF-α for 3–4 h increased the phosphorylation of LPL on Ser 5 and Ser 7, and the actin-bundling process was necessary to form NSZs. Furthermore, we showed that the TNFR1/TRAF-6 pathway could be activated in response to the TNF-α stimulus. Others have demonstrated that TRAF-6 present in the cytoplasm is translocated to the cell membrane following the interaction of TNF-α and TNFRs. TNFRs directly bind through the TRAF-interacting motifs and recruit TRAFs to the cell membrane [73,74,75,76,77]. TRAF-6–Src complex formation was shown to form with the Akt/PKB signaling complex in response to TNF family member stimulation of osteoclast differentiation and activation [31]. Knockouts of TRAF-6 and c-Src are osteopetrotic [35,36,71]. The bone defects in these animals are not due to a lack of osteoclasts but are caused by defective osteoclast activity. Studies have shown that cytoplasmic Src in osteoclasts rapidly colocalizes with TRAF-6 following IL-1 treatment [37]. Consistently, immunostaining analysis demonstrated the diffuse distribution of TRAF-6 and patchy small aggregates closer to the membrane; intracellularly, TRAF-6 is colocalized with Src. These exciting findings allude to the roles of TRAF-6 and Src in osteoclast cytoskeletal organization, actin ring formation, and bone resorption; however, their roles in NSZs formation were not elucidated. Our previous [25] and present studies show that Src inhibition with an inhibitor (PP2) or knockdown of TRAF-6 obstructed LPL-mediated NSZ formation in osteoclasts treated with TNF-α. Thus, TRAF-6 and Src have roles in the early phase of sealing ring organization, i.e., in the actin-bundling process by LPL, independent of integrin αvβ3 signaling.

Phosphorylation of LPL was regulated by PKC (also PKC βII and PKC ζ), PKA, PI3-K, Mst 1 kinase, RSK1, and RSK 2 kinases downstream of the ERK/MAPK pathway [8,39,40,42,78,79,80,81,82,83,84]. Previous studies in our laboratory used the amino-terminal sequence of the LPL peptide ^1^MARGSVSDEE^10^ to inhibit the cellular LPL phosphorylation in osteoclasts [30,56]. The amino acid sequence “RGSV” in the LPL peptide (^1^MARGSVSDEE^10^) matches the Rho kinase consensus RXS/TX. It also has multiple putative kinase recognition motifs for PKC and PKA (SVSD). Peptide sequences’ RXXS/T or RXS/TX’ is known as the phosphorylation consensus sequence of Rho kinase (ROK or ROCK II) in cardiac troponin T and cardiac troponin 1 [85]. PKC has been related to osteoclast survival and function [86,87]. PKCα function was observed as a downstream regulator of αvβ3 integrins in activating cell migration and osteoclastic resorption [88]. Although LPL peptide has a consensus for PKC and PKA kinases, inhibitors of these kinases did not affect TNF-α/TNFR1 regulated phosphorylation of LPL, NSZ formation, and F-actin content in osteoclasts. The response to a stimulus was different for different cell types. Thus, the phosphorylation of LPL may depend on the cell type, function, and signaling cascade.

PI3-K was first identified as a lipid kinase that can bind viral oncoproteins, including v-Src, v-Ros, and polyomavirus middle T antigen. PI3-K has two (N- and C-terminal) SH2 domains and one SH3 domain [89]. The classic SH3 domain arbitrates the interaction of proteins via binding to proline-rich peptides in their respective interacting protein. The preferred binding sequences of Src and PI3-K are XXXRPLPPLPXP and RXXRPLPPLPPP, respectively [90]. Src and TRAF-6 are vital proteins in osteoclast function. The putative SH3-domain-binding motif (PxxPxxP) exclusively found in TRAF-6 is located between the amino acids’ 462–468 domain [31]. Thus, the interaction of TRAF-6 and Src appears to depend on the presence of the Src SH3 domain [31,77]. As shown by others, results obtained with the GST-pull-down studies revealed the pull-down of TRAF-6 with the SH2 and SH3 domains of Src and PI3-K, respectively. Neither TRAF-6 nor LPL was pulled down with the SH2 domains (C- and N-terminal) of PI3-K. The SH2 domains of Lck and Src proteins have a similar affinity and specificity [91]. Therefore, although Lck is not involved in the phosphorylation of LPL, the SH2 domain of Lck was used to determine the binding specificity of TRAF-6 with the SH2-domain. This initial characterization suggests that the interaction between proteins occurs in a sequence-specific manner. However, this study had limitations, and several important questions remain to be evaluated. Although LPL and TRAF-6 are coprecipitated with the SH3 and SH2 domains of PI3-K and Src, the actual order of the interaction requires further elucidation. Therefore, future studies will be focused on this aspect.

## 5. Conclusions

The current study showed that a complex interaction between TNFR1 and signaling proteins regulated the phosphorylation of LPL. The Erk/MAPK pathway was determined as one of the signal-transduction pathways leading to the phosphorylation of LPL [61,83]. Although PKC and PKA regulated phosphorylation of LPL in other cell types, these kinases were not observed as downstream targets of the TNF-α/TNFR1 cascade in osteoclasts. TRAF-6 autoubiquitination was shown to be critical for osteoclast differentiation and function [92]. Studies provided here present insights into the role of Src/PI3-K-mediated TRAF-6 phosphorylation on LPL-mediated regulation of sealing-ring formation and bone resorption. The signal transduction track of TNF-α/TNFR1 involves Src–PI3-K–TRAF-6–Rho/Rho-kinase, which seems appropriate to regulate LPL phosphorylation required for the actin-bundling process and NSZ formation during bone resorption (Figure 6, schematic diagram). LPL phosphorylation and NSZ formation occurred exclusively by the TNF-α/TNFR1 cascade, independently of integrin αvβ3 signaling. The unraveling of the signaling cascade that regulates LPL phosphorylation and NSZ formation represents an essential step in identifying the possible molecular mechanisms fundamental to the sealing-ring formation and bone resorption by osteoclasts. The present study provides further evidence for the interaction of TRAF-6 with the SH3 domain-containing signaling protein. These studies suggest that novel peptidomimetic therapeutic compounds targeting the SH2/SH3 domains may be essential for treating diseases that exhibit osteoclast activation and bone loss.

## Data Availability

Data are available upon request.

## Supplementary Materials

The following are available online at https://www.mdpi.com/article/10.3390/cells10092432/s1, Figure S1: Immunoblotting analysis of the effect overexpression of constitutively active (CA) and kinase defective (KD) Src on the phosphorylation of LPL, Figure S2: Immunoblotting (IB) analysis of the effect TAT-Rho (V14 and N19) on the phosphorylation of LPL, Figure S3: Analysis of the effect of inhibitors to Rho, Rho-kinase, and PI3-K on TRAF-6 phosphorylation, Figure S4: Analysis of actin distribution and F-actin content in resorbing osteoclasts subjected to various treatments.
