# Supplementary material for: L-Plastin Phosphorylation: Possible Regulation by a TNFR1 Signaling Cascade in Osteoclasts
Source: Cells. 2021 Sep 15;10(9):2432. doi: 10.3390/cells10092432 (PMC8464874; doi:10.3390/cells10092432)
Supplement: Supplementary file 1 [file cells-10-02432-s001.zip › cells-1321858-supplementary.pdf]

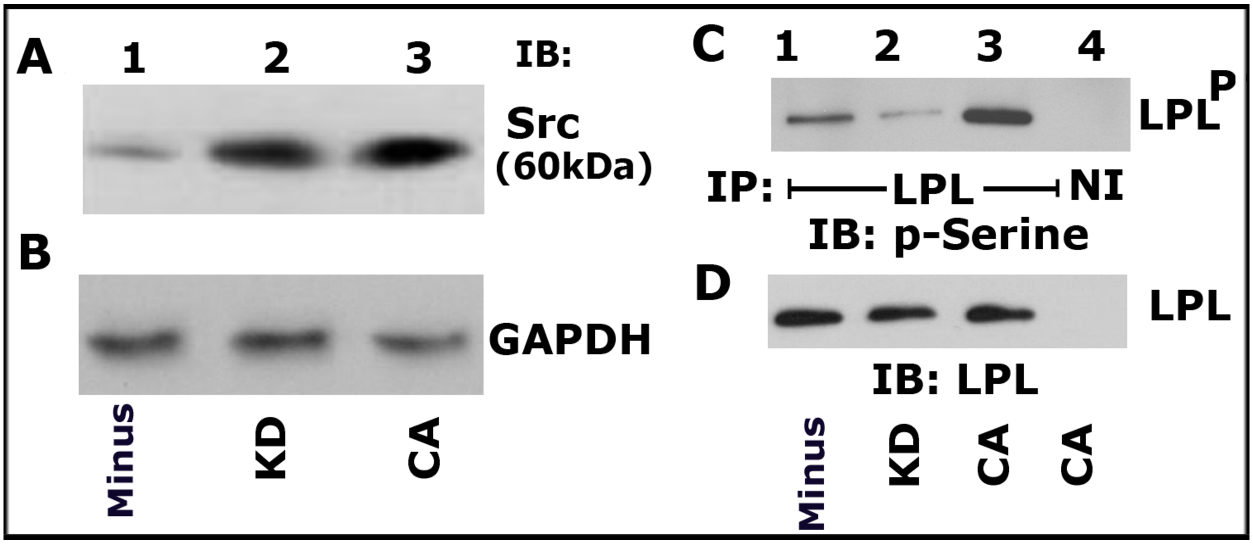

Figure S1. Immunoblotting analysis of the effect overexpression of constitutively active (CA) and kinase defective (KD) Src on the phosphorylation of LPL

Osteoclasts untransfected Src constructs but treated with TNF-α, and bone particles for 3–4h were used as controls and are indicated as minus. Osteoclasts transfected with Src constructs were treated with TNF-α and bone particles for 3–4h. (**A** and **B**) Immunoblotting analysis was done with an Src antibody to determine cellular or endogenous Src (panel A, lane 1) and transfected Ad-Src (CA and KD; lanes 2 and 3) expression levels. The blot was stripped and immunoblotted with a GAPDH antibody (B). (**C** and **D**) IB analysis with a p-Serine antibody. Lysates made from osteoclasts untransfected or transfected with Src constructs and subsequently incubated with TNF-α and bone particles for 3–4 h were used to detect LPL phosphorylation (C and D). First, immunoprecipitates made with an LPL antibody (A, 1–3) and a non-immune (G, lane 4) were immunoblotted with a p-Serine antibody; subsequently, the blot was stripped and immunoblotted with an antibody to LPL to determine the immunoprecipitated levels of LPL (B). Immunoprecipitation done with a non-immune serum is used to control immunoprecipitation. Experiments were repeated three to four times. Uncropped images for these results are below

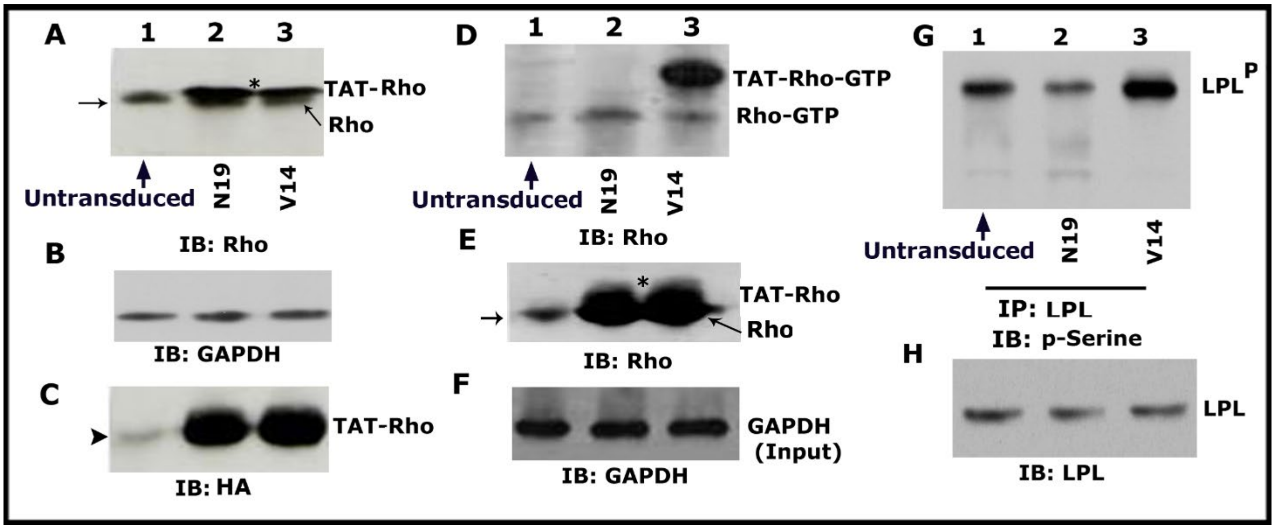

Figure S2. Immunoblotting (IB) analysis of the effect TAT-Rho (V14 and N19) on the phosphorylation of LPL. Osteoclasts not transduced with Rho proteins were used as controls and are indicated as untransduced. Arrows in panels A and E point to endogenous (cellular) Rho protein (~25–28 kDa), and an asterisk between lanes 2 and 3 (panels A and E) denotes the transduced TAT-Rho (V14 and N19; ~35–37 kDa) proteins. For results shown in panels D and G, lysates made from osteoclasts untransduced or transduced with TAT-Rho proteins and subsequently incubated with TNF-α and bone particles for 3–4 h were used to detect Rho activity (D) and LPL phosphorylation (G). (**A–C**) Lysates made from osteoclasts untransduced or transduced with TAT-Rho proteins (lanes 2 and 3) were immunoblotted with a Rho antibody. Blot was sequentially stripped and immunoblotted with an antibody to GAPDH (B) and HA (C). Arrowhead indicates overflowing of sample into lane 1 from lane 2. (**D–F**) Analysis of binding of GTP-bound to GST fused RBD beads. Lysates made from osteoclasts treated as indicated in panel D were incubated with GST-RBD coupled glutathione beads. Bound proteins were immunoblotted with an antibody Rho (D). IB analysis of the total lysate with a Rho antibody demonstrates endogenous and transduced Rho proteins (E). In addition, direct IB of the lysates with a GAPDH antibody in panel F indicates the total protein (Input) used for GST-RBD binding assay. In panel D, Rho-GTP and TAT-Rho-GTP represent the cellular Rho and the transduced V14 Rho bound to GTP. (**G** and **H**) Analysis of the effect of Rho transduction on LPL phosphorylation. Lysates made from osteoclasts untransduced or transduced with TAT-Rho proteins (lanes 2 and 3) were immunoprecipitated with an LPL antibody. Then, IB was done with a p-Serine antibody (G), and the blot was immunoblotted with an antibody to LPL after stripping. Experiments were repeated three times and obtained similar results. Uncropped images for these results are below.

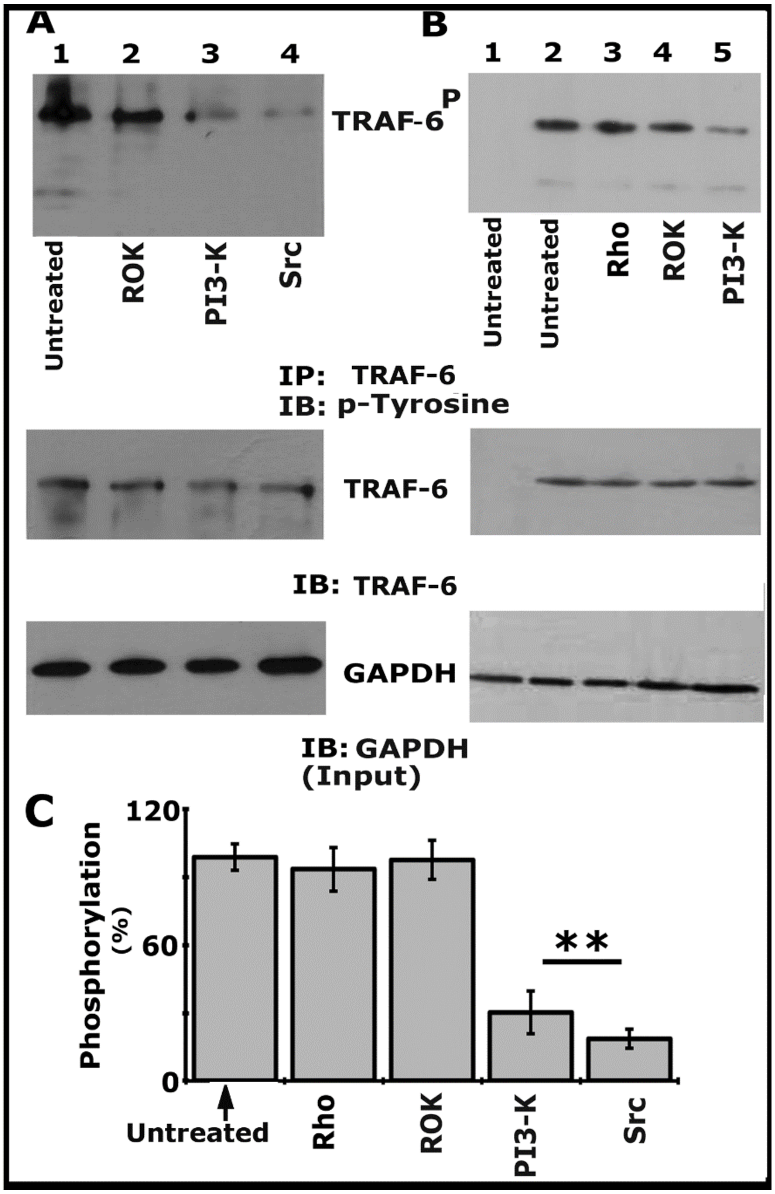

Figure S3. Analysis of the effect of inhibitors to Rho, Rho-kinase, and PI3-K on TRAF-6 phosphorylation (**A–C**) Lysates made from osteoclasts treated with inhibitors to PI-3K (wortmannin), Rho kinase (Y27632), Src (PP2), and Rho (C3 exoenzyme) were immunoprecipitated with an antibody to TRAF6 (panels A and B) or non-immune serum (Panel B, NI; lane 1) for IB analysis with a p-Tyrosine antibody (Top panels in A and B). Subsequently, the blot was stripped and immunoblotted with an antibody to TRAF6 to determine immunoprecipitated levels of TRAF6 (middle panel in A and B). Direct immunoblotting of the lysates with a GAPDH antibody in panel G indicates the total protein (Input) used for immunoprecipitation with TRAF6 (Bottom panel in A and B). Results represent two of the three experiments with similar findings. Percent phosphorylation of TRAF-6 is provided as a graph (C). ** $p < 0.01$ versus untreated osteoclasts. Uncropped images for these results are below.

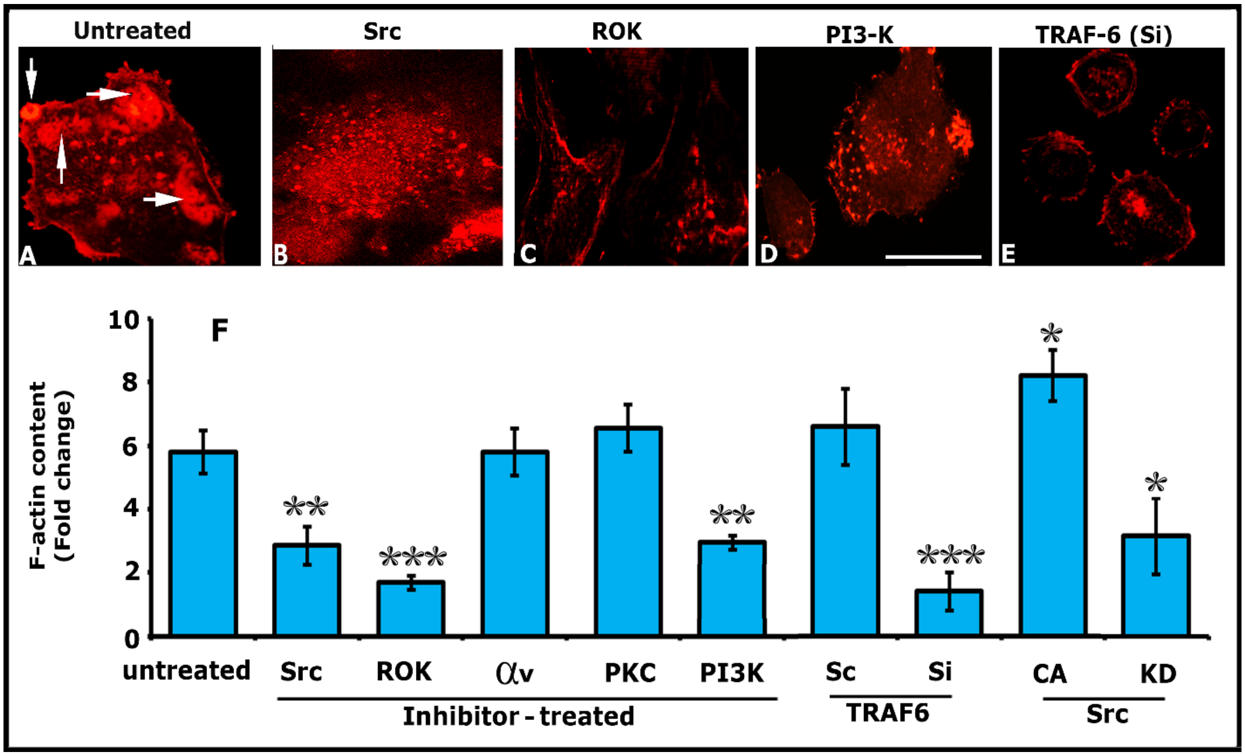

Figure S4. Analysis of actin distribution and F-actin content in resorbing osteoclasts subjected to various treatments. (**A**–**E**) Osteoclasts cultured on dentine slices for 3–4 h were treated as indicated in the figure in the presence of TNF-α. Then confocal analysis was done in these osteoclasts after staining with rhodamine-phalloidin to determine actin distribution; white arrows in A point to nascent sealing zones. Scale bar: 100 μm. Results represent one of the two experiments performed with similar results. (**F**) Measurement of F-actin content by rhodamine-phalloidin binding to osteoclasts treated as indicated in the panel. The F-actin content of the 0-min cells was assigned a value of 1.0, and all other values were expressed relative to the 0-min values. Values plotted are mean ± SEM from three experiments ***$p < 0.001$; **$p < 0.01$, versus untreated osteoclasts. Results represent one of the three experiments performed with similar results.

Uncropped Images

Figure 1 The effect of SiRNA to TRAF 6 on LPLphosphorylation

A and B:

A Top middle GAPDH

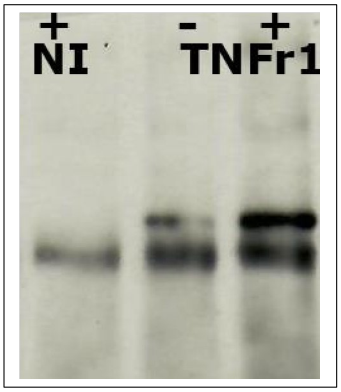

IP TNFR1;

IB-p-Tyr

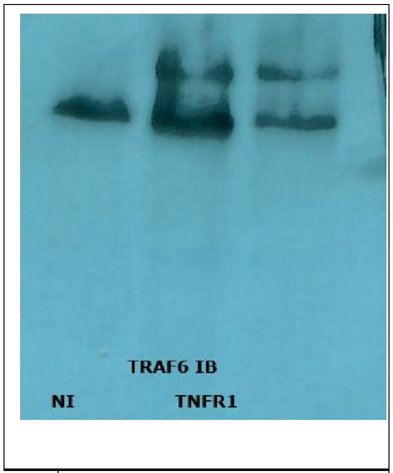

IB: TRAF-6

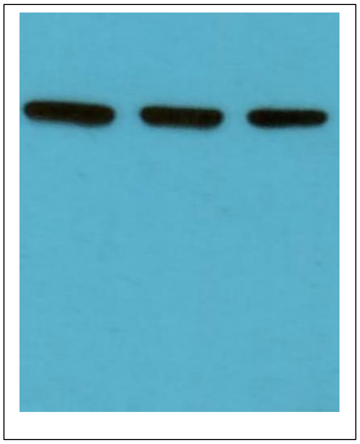

Total input GAPDH

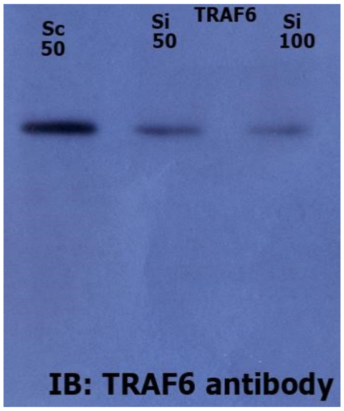

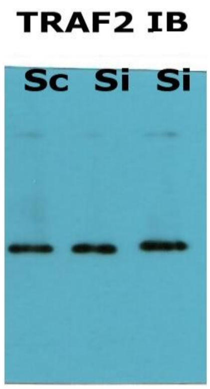

B **Top** **GAPDH**

TRAF 6 levels in SiRNA treated cells; IB: TRAF6

TRAF 6 Si RNA effect IB: TRAF2

Figure 1C TRAF6 siRNA effect on LPL phosphorylation

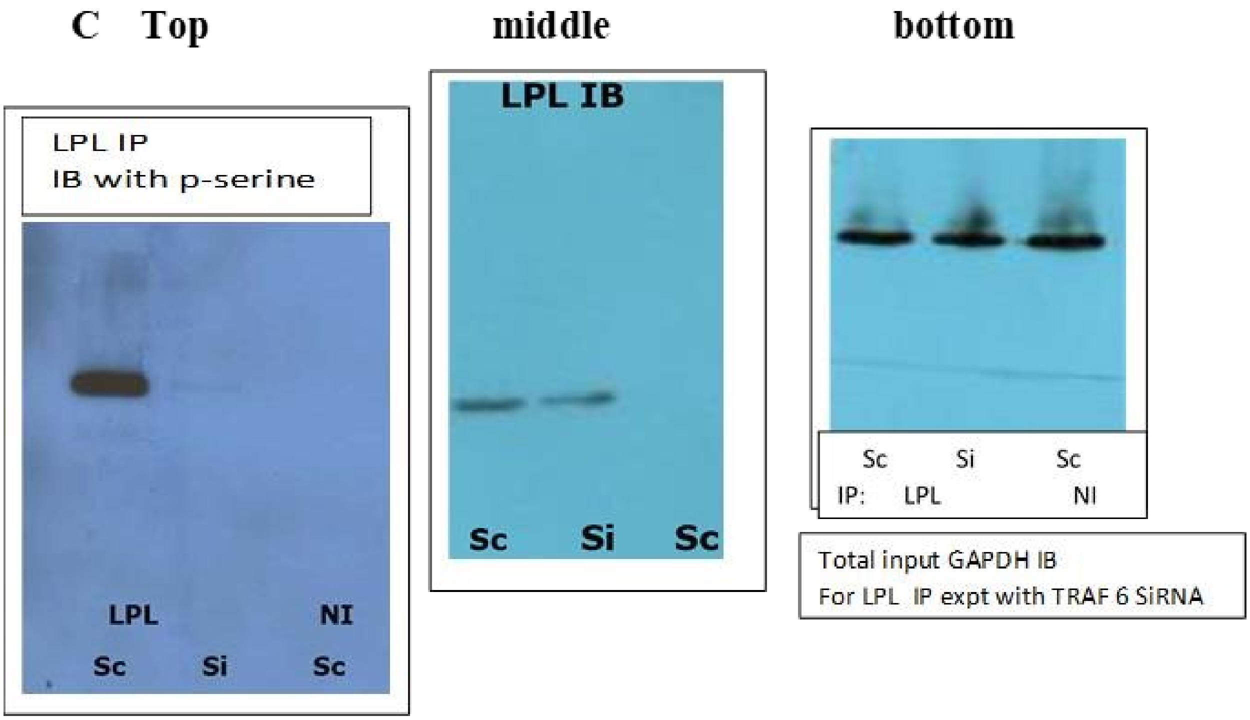

Figure 2

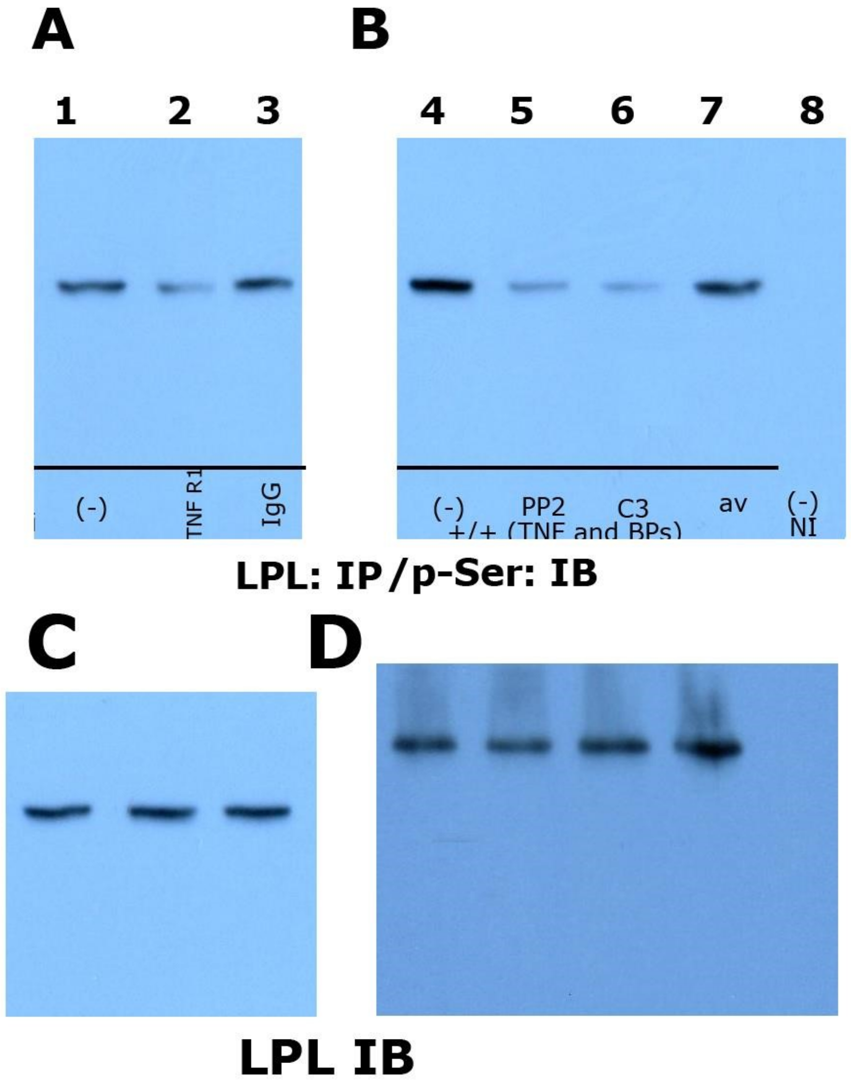
A
B
1
2
3
4
5
6
7
8
(-)
TNF R1
IgG
(-)
PP2
C3
av
(-)
NI
+/+ (TNF and BPs)
LPL: IP/p-Ser: IB
C
D
LPL IB

Figure 4

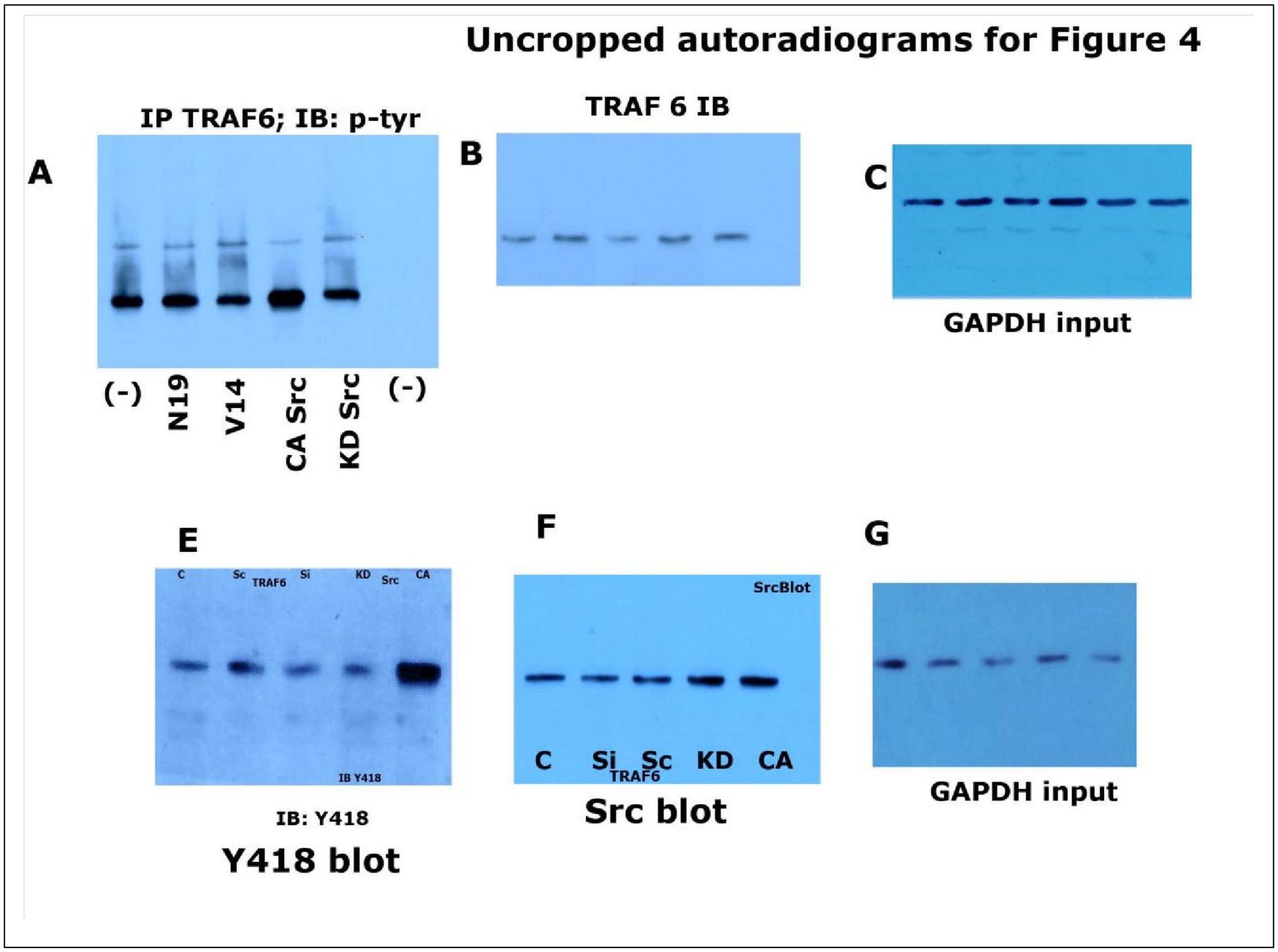
Uncropped autoradiograms for Figure 4
IP TRAF6; IB: p-tyr
A
(-)
N19
V14
CA Src
KD Src
(-)
TRAF 6 IB
B
C
GAPDH input
E
C
Sc
TRAF6
Si
KD
Src
CA
IB Y418
IB: Y418
Y418 blot
F
SrcBlot
C
Si
Sc
TRAF6
KD
CA
Src blot
G
GAPDH input

Uncropped autoradiograms for Supplementary Figure S1 and S2

Supplementary Figure S1

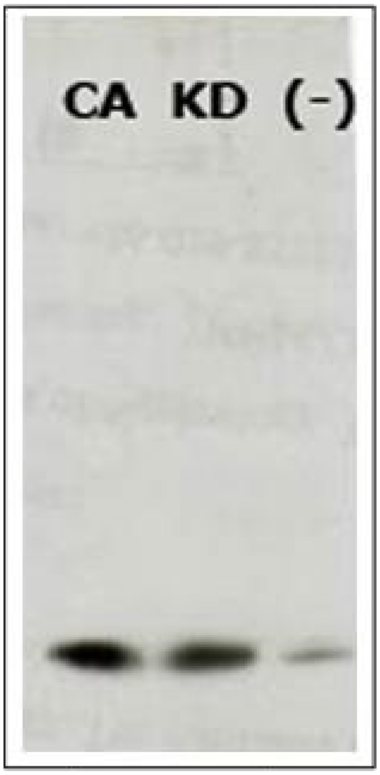

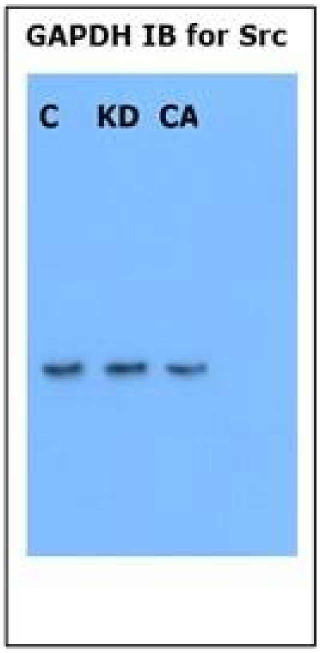

GAPDH IB

Src IB

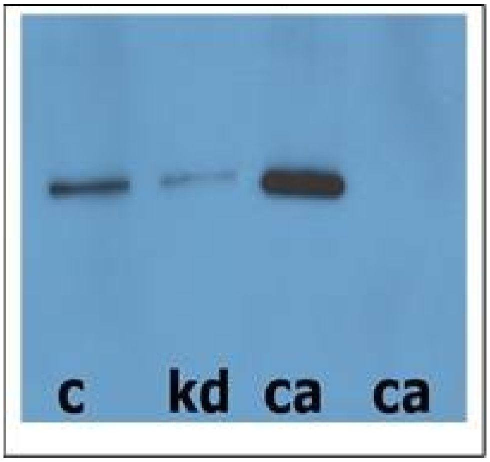

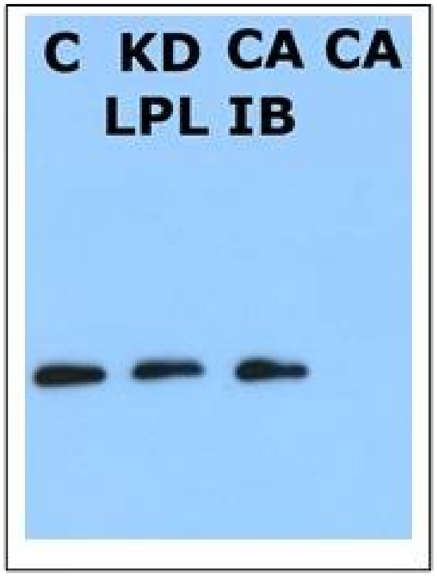

Supplementary Figure S2 A-F

1. **Rho Transduction and LPL phosphorylation**

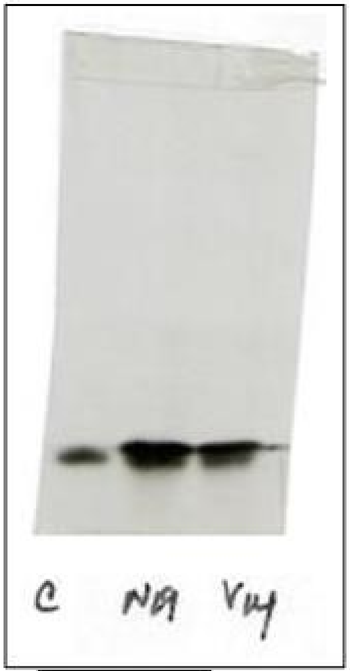

IB with Rho

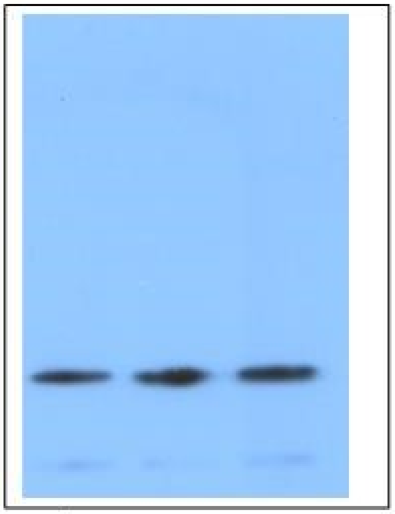

IB with GAPDH

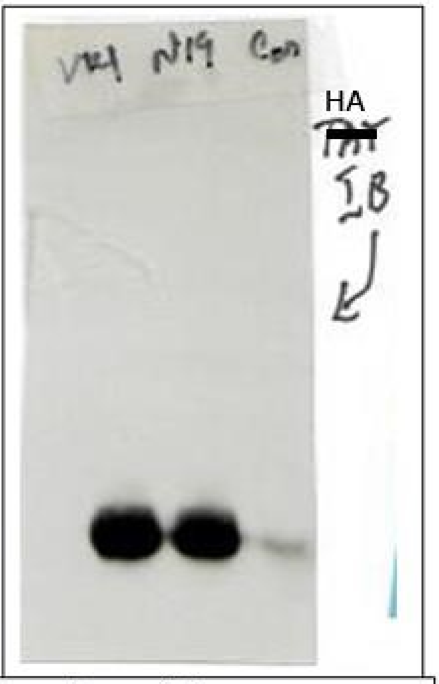

IB with HA antibody

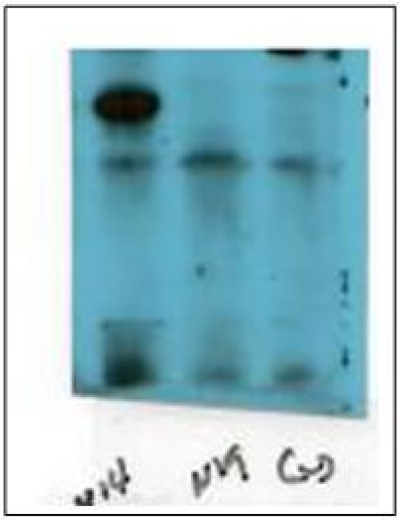

GST- pulldown assay and IB with Rho
Pull down with GST-fused rhotekin

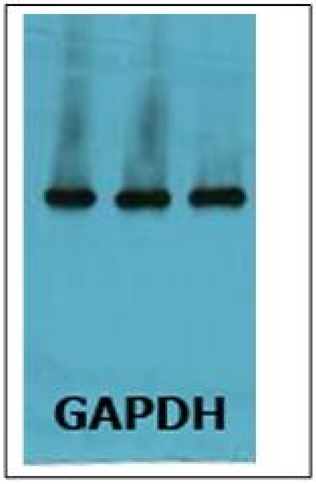

Total input GAPDH IB

Figure S2- Panels G and H

H

(-) N19 V14

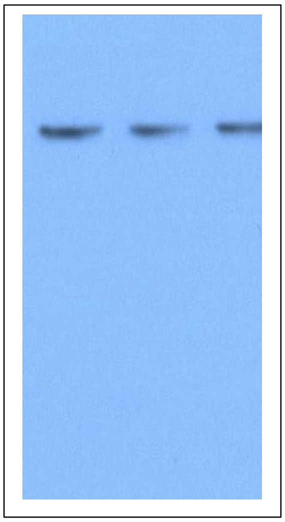

IB with LPL

G

(-) N19 V14

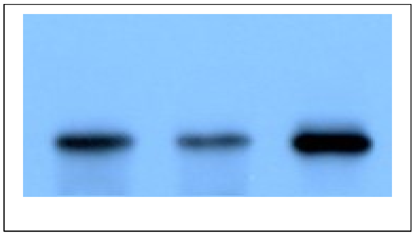

LPL IP
IB with p-serine

Figure S3

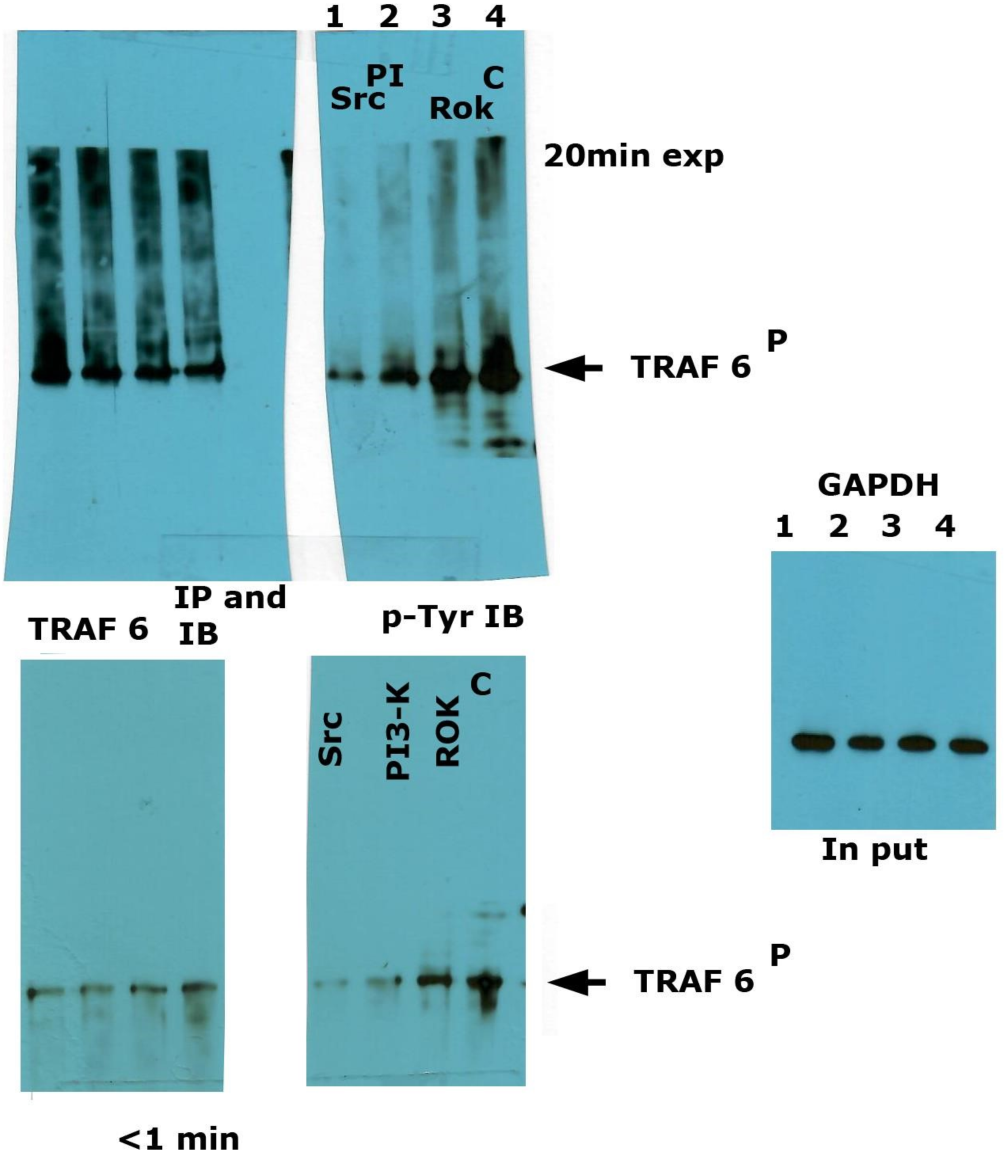

1 2 3 4
Src
PI
Rok
C
20min exp
TRAF 6 P
GAPDH
1 2 3 4
TRAF 6
IP and IB
p-Tyr IB
Src
PI3-K
ROK
C
In put
TRAF 6 P
<1 min
